# Blood transcriptional signatures for tuberculosis testing

**DOI:** 10.1016/S2213-2600(20)30045-X

**Published:** 2020-03-13

**Authors:** Simon C Mendelsohn, Stanley Kimbung Mbandi, Mark Hatherill, Thomas J Scriba

**Affiliations:** South African Tuberculosis Vaccine Initiative, Institute of Infectious Disease and Molecular Medicine and Division of Immunology, Department of Pathology, University of Cape Town, Observatory, Cape Town, 7925, South Africa

Case-finding strategies for tuberculosis diagnosis rely on symptom screening, which is associated with poor sensitivity,^1^ resultant delays in diagnosis, increased patient morbidity, and ongoing transmission. There is need for a more sensitive, non-sputum-based triage test to exclude tuberculosis at the primary care level, and for mass screening in high-burden settings.^2^ Several blood transcriptional diagnostic signatures have been described;^3^ however, these were invariably discovered and validated in carefully selected case-control cohorts, inflating diagnostic accuracy. A crucial step in the development pathway is assessment of prospective diagnostic accuracy in real-world health-care settings.

In *The Lancet Respiratory Medicine*, Carolin Turner and colleagues^4^ present the first prospective, systematic head-to-head comparison of the diagnostic accuracy of 27 blood transcriptional signatures. These were identified from their previous systematic review. From 181 symptomatic adults who presented to a primary health-care clinic in South Africa, Turner and colleagues obtained blood for RNA sequencing and sputum for culture and molecular testing using Xpert MTB/RIF (Xpert) and Xpert MTB/RIF Ultra (Ultra), and compared the diagnostic accuracy of the candidate signatures with that of culture or Xpert for active tuberculosis.

Notably, no signatures met the WHO target product profile^5^ benchmark criteria for a non-sputum confirmatory tuberculosis diagnostic test (minimum 65% sensitivity, 98% specificity) or optimum criteria for a tuberculosis triage test (95% sensitivity, 80% specificity). The four best-performing signatures had similar diagnostic accuracy, independent of HIV status, and met or approached the minimum WHO criteria for a tuberculosis triage test. These results suggest that transcriptional signature diagnostic performance might have peaked and it is unlikely that new transcriptional signatures discovered in existing case-control cohorts will have better diagnostic accuracy than existing signatures. It might also prove challenging to reproduce the results achieved using batch-corrected, high-throughput methods, such as RNA sequencing, when signatures are translated to point-of-care RNA quantitation technologies in a real-world application.

Tuberculosis is a spectrum that spans quiescent, latent infection through to subclinical and active symptomatic disease.^6^ The apparent ceiling and poor performance of some signatures in field settings, as tested by Turner and colleagues,^4^ might be partly accounted for by discovery methods that have relied on feature selection and model construction to achieve binary differentiation between homogenous groups that represent the extremes of the spectrum. Given the difficulty in diagnosing subclinical (asymptomatic, sputum culture-positive) and paucibacillary (sputum smear-negative, culture-positive) tuberculosis, a latent class modelling approach could be considered in future tuberculosis discovery and validation studies, to account for uncertainty in disease classification.^7^

Another consideration is that transcriptional signatures measure expression of non-specific inflammation, primarily comprised of interferon-stimulated genes (ISG). Because conditions other than tuberculosis, such as viral infections, induce ISG expression, false-positive results are inevitable in some symptomatic individuals without tuberculosis. A heterogenous prospective discovery cohort that is representative of the target population, in this case symptomatic clinic attendees, might result in more tuberculosis-specific gene selection and enhance downstream performance. A multinomial or multilabel classification model, in which individuals can be assigned to one or more outcomes, is an alternative strategy.^8^ Finally, integration of clinical variables might also provide further incremental improvements in diagnostic accuracy. Whether new biomarker discovery efforts in large, multicentre, prospective cohorts of unselected symptomatic patients seeking health care will lead to better performance remains to be seen.

A key objective of tuberculosis triage tests is to rapidly screen individuals seeking care to rule out those without disease, thereby reducing the volume of more expensive confirmatory diagnostic tests.^2^ The price of Xpert cartridges is currently fixed at US$9·98; however, it is likely that the cost of molecular testing will soon drop. It is imperative that a tuberculosis triage test should cost less than confirmatory testing, ideally closer to $2 after scale-up, as proposed by expert consensus.^5^ Whether a point-of-care transcriptional biomarker-based test could achieve the $2 price point, and be sufficiently high-throughput (>10 tests per 6 h, <1 h per test) will need to be ascertained.^5^ Given that multiple simple, fast, and affordable non-sputum-based tuberculosis screening tools have been developed, including C-reactive protein-based screening^9^ and computer-aided radiographic detection, the impetus to take host transcriptional biomarkers to market might diminish.

Ultra, which offers improved sensitivity over the previous Xpert test^10^ and other similar tests, might also supplant the need for a triage test, especially in tuberculosis high-burden settings, such as the one studied by Turner and colleagues,^4^ where almost one in three symptomatic adults had microbiologically confirmed disease. However, with greater sensitivity comes a loss in specificity,^10^ probably a consequence of detecting non-viable mycobacterial DNA, a remnant of previous tuberculosis disease, or detection of early paucibacillary disease, which might self-cure. Turner and colleagues^4^ found that transcriptional signatures correctly characterised Ultra-positive, culture-negative (false-positive) sputum samples, improving test specificity. This finding raises the possibility of repurposing transcriptional or other host-response biomarkers as adjunctive tests to measure *Mycobacterium tuberculosis* activity in Ultra-positive individuals with previous tuberculosis disease, or with trace results, to rule-out false-positives or to identify those who do not have active disease but are at high risk of disease progression.

Further large-scale, prospective validation studies, ideally using RNA quantitation technologies compatible with existing point-of-care platforms, to compare multiple transcriptional signatures in real-world settings and across multiple epidemiological and geographical locations, are therefore important to advance transcriptional signatures through the diagnostics pipeline.
